# Decomposing socioeconomic inequality in dental caries in Iran: cross-sectional results from the PERSIAN cohort study

**DOI:** 10.1186/s13690-020-00457-4

**Published:** 2020-08-18

**Authors:** Farid Najafi, Satar Rezaei, Mohammad Hajizadeh, Moslem Soofi, Yahya Salimi, Ali Kazemi Karyani, Shahin Soltani, Sina Ahmadi, Enayatollah Homaie Rad, Behzad Karami Matin, Yahya Pasdar, Behrooz Hamzeh, Mehdi Moradi Nazar, Ali Mohammadi, Hossein Poustchi, Nazgol Motamed-Gorji, Alireza Moslem, Ali Asghar Khaleghi, Mohammad Reza Fatthi, Javad Aghazadeh-Attari, Ali Ahmadi, Farhad Pourfarzi, Mohammad Hossein Somi, Mehrnoush Sohrab, Alireza Ansari-Moghadam, Farhad Edjtehadi, Ali Esmaeili, Farahnaz Joukar, Mohammad Hasan Lotfi, Teamur Aghamolaei, Saied Eslami, Seyed Hamid Reza Tabatabaee, Nader Saki, Ali Akbar Haghdost

**Affiliations:** 1 Research Center for Environmental Determinants of Health, Health Institute, Kermanshah University of Medical Sciences, Kermanshah, Iran; 2 School of Health Administration, Faculty of Health, Dalhousie University, Halifax, Canada; 3 Social Development and Health Promotion Research Center, Kermanshah University of Medical Sciences, Kermanshah, Iran; 4 Department of Social Welfare Management, University of Social Welfare and Rehabilitation Sciences, Tehran, Iran; 5 Social Determinants of Health Research Center, Guilan University of Medical Sciences, Rasht, Iran; 6 Department of Health Information Technology, Paramedical School, Kermanshah University of Medical Sciences, Kermanshah, Iran; 7 Liver and Pancreatobiliary Diseases Research Center, Digestive Diseases Research Institute, Tehran University of Medical Sciences, Tehran, Iran; 8 Department of Anesthesiology, Sabzevar University of Medical Sciences, Sabzevar, Iran; 9 Noncommunicable Diseases Research Center, Fasa University of Medical Sciences, Fasa, Iran; 10 Gastroenterohepatology Research Center, Shiraz University of Medical Sciences, Shiraz, Iran; 11 Social determinants of Health Research Center, Urmia Jundishapur University of Medical Sciences, Urmia, Iran; 12 Modeling in Health Research Center, Shahrekord University of Medical Sciences, Shahrekord, Iran; 13 Digestive Disease Research Center, Ardabil University of Medical Sciences, Ardabil, Iran; 14 Liver and Gastrointestinal Diseases Research Center, Tabriz University of Medical Sciences, Tabriz, Iran; 15 Diabetes Research cente, Mazandaran University of Medical Sciences, Sari, Iran; 16 Health Promotion Research Center, Zahedan Jundishapur University of Medical Sciences, Zahedan, Iran; 17 Department of Cardiology, Medical school, Rafsanjan University of Medical Sciences, Rafsanjan, Iran; 18 Gastrointestinal and Liver Diseases Research Center, Guilan University of Medical Sciences, Rasht, Iran; 19 Shahid Sadoghi University of Medical Sciences, Yazd, Iran; 20 Department of Public Health, School of Public Health, Hormozgan University of Medical Sciences, Bandar Abbas, Iran; 21 Pharmaceutical Research Center, Pharmaceutical Research Institute, Mashhad University of Medical Sciences, Mashhad, Iran; 22 Research Center for Health Sciences, Shiraz University of Medical Sciences, Shiraz, Iran; 23 Hearing Research Center, Ahvaz Jundishapur University of Medical Sciences, Ahvaz, Iran; 24 Modeling in Health Research Center, Institute for Futures Studies in Health, Kerman University of Medical Sciences, Kerman, Iran

**Keywords:** Socioeconomic status, Dental caries, Concentration index, Decomposition, Iran

## Abstract

**Background:**

The current study aimed to measure and decompose socioeconomic-related inequalities in DMFT (decayed, missing, and filled teeth) index among adults in Iran.

**Methods:**

The study data were extracted from the adult component of Prospective Epidemiological Research Studies in IrAN (PERSIAN) from 17 centers in 14 different provinces of Iran. DMFT score was used as a measure of dental caries among adults in Iran. The concentration curve and relative concentration index (RC) was used to quantify and decompose socioeconomic-related inequalities in DMFT.

**Results:**

A total of 128,813 adults aged 35 and older were included in the study. The mean (Standard Deviation [SD]) score of D, M, F and DMFT of the adults was 3.3 (4.6), 12.6 (10.5), 2.1 (3.4) and 18.0 (9.5), respectively. The findings suggested that DMFT was mainly concentrated among the socioeconomically disadvantaged adults (RC = − 0.064; 95% confidence interval [CI), − 0.066 to − 0.063). Socioeconomic status, being male, older age and being a widow or divorced were identified as the main factors contributing to the concentration of DMFT among the worse-off adults.

**Conclusions:**

It is recommended to focus on the dental caries status of socioeconomically disadvantaged groups in order to reduce socioeconomic-related inequality in oral health among Iranian adults. Reducing socioeconomic-related inequalities in dental caries should be accompanied by appropriate health promotion policies that focus actions on the fundamental socioeconomic causes of dental disease.

## Background

Dental caries is one of the major public health concerns throughout the world. Poor oral health condition adversely affects the quality of life, oral health status and well-being of people [1]. Dental caries can potentially lead to social and psychological problems. Besides the negative health consequences, poor oral health condition, high prevalence of oral disorders imposes a substantial financial burden to individuals, their families, as well as to the society as a whole [2, 3].

Socioeconomic-related inequalities in various health outcomes constitute a main challenge for public health [4–6]. According to the World Health Organization’s Commission on Social Determinants of Health (CSDH), health inequalities are the result of the exposure to health risks among those living in socioeconomically disadvantaged circumstances [7, 8]. Previous studies highlighted the significant negative association between socioeconomic status (SES) and dental caries [9, 10]. The existing literature [9–13] indicated widespread inequalities in oral health outcomes across socioeconomic groups both in developed and developing countries. Higher SES also positively associated with cleaning the teeth more effectively and frequently and with using more oral hygiene aids [14].

Although dental caries rates in the developed world are decreasing [11, 15–18], data from developing countries shows that high dental caries continues to be a public health problem [19–22]. While there is data from Iran that shows a similar trend in dental caries, little is known about the impact of SES on dental caries [4, 17]. Using information available in the Prospective Epidemiological Research Studies in IrAN (PERSIAN), in this cross-sectional analysis, we aimed to measure socioeconomic inequalities in dental caries, as measured by DMFT (decayed, missing, and filled teeth) index, among adults (35 years and older) in Iran. Furthermore, we decomposed socioeconomic inequality in DMFT index in order to identify factors explaining socioeconomic inequality in dental caries. The results of our study provide useful information for health care policymakers in Iran as a developing country and are useful for other developing regions in order to design effective interventions to decline inequality in oral disorders among Iranian adults.

## Methods

### Study setting

Iran, as a developing country, is located in the Eastern Mediterranean Region with an area of 1,648,000 km sq. Based on the 2016 census data, the total population of Iran was about 80 million people.

### Data source and variables

In this cross-sectional study, we extracted and merged the required data from the adult component of the PERSIAN, which has been launched by the Ministry of Health and Medical Education (MoHME) to collect epidemiological data from 17 centers in 14 different provinces of Iran, since 2014, as follows: Kermanshah (KSH), Gilan (GI), Fars (FA), East Azerbaijan (EA), Mazandaran (MA), Sistan and Balouchestan (SB), Yazd (YA), Kerman (KE), Khouzestan (KH), Chaharmahal and Bakhtiari (CB), Hormozgan (HO), West Azerbaijan (WA), Ardabil (AR) and Razavi Khorasan (RK). While there is only one PERSIAN cohort center in 13 provinces, FA and RK have three (Fasa, Kavar and Kharameh) and two centers (Sabzevar and Mashhad), respectively. We obtained data from all the centers. The characteristics of cohort centers used in the study showed in Appendix. Finally, after excluding the subjects with the missing values in the variables included in the study, a total of 128,813 adults, aged 35 years and above, from 14 provinces of Iran were included in the analysis.

The cohort questionnaire consisted of three parts of general, medical and nutrition with 482 questions. The first part included general questions on demographics, SES, lifestyle, occupational history, physical activity, sleep and circadian rhythm and mobile use. The second part consisted of questions related to medical issues (past and present medical history, type of treatment, blood pressure and pulse measurements and oral health). The third part asked questions regarding personal habits questions such as smoking, drinking alcohol and drug use. The cohort questionnaire was administrated by trained interviewers. Quality assurance (QA) and quality control (QC) measures were re-checked by the central and local QA/QC teams to ensure all procedures are performed in accordance with the PERSIAN Cohort protocol. The PERSIAN cohort is a cohort study that has different studying sites around Iran. Because of the coordination among these cohorts, the data collection tools and their definitions were comparable; therefore, we could compile their datasets with minimum conflicts. More details about the PERSIAN study can be found elsewhere [23, 24].

The outcome variables was DMFT index as a measure of dental caries in the study [25]. The DMFT score was measured as the total number of teeth that are decayed (D), missed (M) and filled (F) teeth; thus the mean of DMFT for the total samples is calculated by dividing the sum of all the DMFT scores by the total number of samples [4, 26]. The DMFT index is calculated using a medisporex catheter by direct examination of teeth. To correct examination, samples and the trainer students sat close to the window to perform the examination under the maximum natural light. Then, recoded results recheck by a medical doctor on individuals. As per current literature [4, 11, 27–29], we used a wide variety of demographics (e.g., age groups, sex and marital status), unhealthy behaviors (e.g., alcohol drinking and smoking status), SES (e.g., level of education, durable assets, and housing characteristics) and place of residence (cohort site or province) as determinants of DMFT in the decomposition analysis.

### Statistical analysis

#### Measuring socioeconomic status

Principal component analysis (PCA) technique [30, 31] was used to construct SES of samples. We entered those assets and housing characteristics (e.g., having car, motorcycle, bicycle, refrigerator, freezer, radio, stove, vacuum machine, personal computer, CD/DVD player, sewing machine, cooler, washing mashing, microwave, central heating, having kitchen, bathroom, use of natural gas for cooking, per capita house area per capita rooms and access to piped drinking water, electricity, telephone, internet and sewage network) and education level in the PCA. Based on the socioeconomic scores, samples were divided into five SES groups (quintiles), from poorest to richest.

#### Measuring and decomposing socioeconomic inequality in Oral health

We used both the concentration curve and relative concentration index (RC) to quantify and decompose socioeconomic-related inequalities in DMFT among Iranian adults (35 years and older) in the 14 provinces combined as well as in each province, separately. The RC is calculated based on the concentration curve, which graphs the cumulative percentage of participants ranked by SES (the constructed SES scores) on the x-axis and the cumulative percentage of a health variable of interest (DMFT score) on the y-axis. The RC is equivalent to twice the area between the line of perfect equality (45-degree line) and concentration curve. The values of the RC range from − 1 to + 1. If the concentration curve lies under (above) the line of perfect equality, the sign of the RC is positive (negative). The negative value of the RC indicated that DMFT score is more concentrated among rich vice versa. The value of zero suggested perfect equality [32].

The following formula was used to calculate the RC:

1 $$ 2{\sigma}_r^2\left(\frac{y_i}{\mu}\right)=\upalpha +\upvarphi {r}_i+{\varepsilon}_i $$

Where *μ* shows the mean of the outcome variable of interest (i.e., DMFT scores) for the whole sample; *y*_*i*_ presents the outcome variable (DMFT score) for individual *i*; and *r*_*i*_ is the fractional rank in the SES distribution for individual *i*; ($$ {r}_i=\raisebox{1ex}{$i$}\!\left/ \!\raisebox{-1ex}{$n$}\right. $$, where is n is the rank of individual *i* based on the SES in the sample of *n*); and $$ 2{\sigma}_r^2 $$ denotes the variance of fractional rank. The (OLS) estimate of φ is the RC [33].

The RC was decomposed to identify the main factors that contributed to the observed socioeconomic inequality in DMFT in the 14 Iranian provinces included in the study. Consider the following linear regression model that links DMFT score, *y*, to a set of *k* explanatory factors ,*x*_*k*_:

2 $$ y=\alpha +{\sum}_k{\beta}_k{x}_k+\varepsilon $$

Wagstaff et al. [34] showed that the RC can be decomposed to its determinants using the following formula:

3 $$ RC={\sum}_k\left(\frac{\beta_k{\overline{x}}_k}{\mu}\right){RC}_k+\frac{G{C}_{\varepsilon }}{\mu } $$

Where ${\overline{x}}_k$ is the mean of explanatory variables, *RC*_*k*_ is the RC for explanatory variables. The $\frac{\beta_k{\overline{x}}_k}{\mu }$ can be defined as the elasticity of the health outcome variable with respect to the explanatory variables. Elasticity shows the amount of change in dependent variable associated with a one-unit change in the explanatory variable. A negative (positive) elasticity for an explanatory variable in our study indicates that an increase in an explanatory variable decreases (increases) the DMFT score. Based on Eq. 3, each of explanatory variable contributes to socioeconomic-related inequality in DMFT if the elasticity of the variable is statistically significant and the variable is unequally distributed by SES. The *GC*_*ε*_ indicates the generalized concentration index for the error term and it reflects socioeconomic-related inequality in DMFT that is not explained by explanatory variables included in the study All data analysis performed by Stata version 14.2 (StataCorp, College Station, TX, USA) and *p*-value less than 0.05 was considered statistically significant.

## Results

### Descriptive statistics

The descriptive statistics of all the variables used in the study are presented in Table 1. Of the total of 128,813 adults aged 35 and older included in the study, 45.5% were males and 55.5% were females. The average age of participants was 49.3 years (standard deviation [SD] = 9.18). A majority of the study population (90.9%) was married. In addition, about 21.7% of the samples were smokers and 9.1% used alcohol in the past year.

**Table 1:** Descriptive statistics of variables used in the study

Variables	n (*N* = 128,813)	Proportion (%)
Demographic variables		
*Age groups*		
35–44	46,112	35.7
45–54	43,329	33.6
55–64	31,150	24.2
65 and older	8222	6.4
*Sex*		
Male	57,346	44.51
Females	71,467	55.49
*Marital status*		
Single	2926	2.27
Married	117,116	90.91
Divorced or widowed	8771	6.82
Socioeconomic status		
1 (Poorest)	25,595	19.87
2	25,703	19.94
3	25,812	20.04
4	25,826	20.09
5 (Wealthiest)	25,877	20.10
Behavioral variables		
*Smoking status*		
Smoker	25,877	21.68
Non-smoker	100,880	78.32
*Drinking alcohol*		
Yes	11,674	9.10
No	117,139	90.90
Region of cohort (province)		
Fars (FA)	22,257	17.28
Guilan (GI)	10,498	8.15
Kermanshah (KSH)	10,040	7.79
East Azerbaijan (EA)	14,927	11.59
Mazandaran (MA)	10,248	7.96
Sistan and Balouchestan (SB)	8208	6.37
Yazd (YA)	9272	7.20
Kerman (KER)	9869	7.66
Khouzestan (KH)	8987	6.97
Chaharmahal and Bakhtiari (CB)	6642	5.16
Hormozgan (HO)	3329	2.58
West Azerbaijan (WA)	3444	2.67
Ardabil (AR)	8180	6.35
Razavi Khorasan (RK)	2921	2.27

The mean (SD) score of D, M, F and DMFT of the adults was 3.3 (4.6), 12.6 (10.5), 2.1 (3.4) and 18.0 (9.5), respectively. There is, however, variation among the provinces in the average score of DMFT score. As reported in Table 2, the average DMFT score was greater in the provinces of East Azerbaijan, Fars, Yazd and Ardabil compared to the rest of the provinces included in the study.

**Table 2:** The mean of D, M, F and DMFT score by total of sample and province

	Decayed (SD)	Missed (SD)	Filled (SD)	DMF (SD)
Fars (FA)	6.7 (6.7)	13.1 (10.0)	1.1 (2.5)	20.9 (9.4)
Guilan (GI)	2.2 (2.5)	10.9 (9.3)	1.5 (2.6)	14.6 (8.8)
Kermanshah (KSH)	3.1 (4.0)	11.7 (9.9)	1.4 (2.6)	16.2 (9.2)
East Azerbaijan (EA)	2.2 (3.6)	16.8 (11.3)	2.3 (3.7)	21.3 (9.0)
Mazandaran (MA)	2.4 (3.4)	12.5 (10.3)	2.4 (3.3)	17.3 (9.1)
Sistan and Balouchestan (SB)	4.1 (4.5)	11.5 (9.1)	1.8 (1.8)	17.4 (9.0)
Yazd (YA)	2.2 (3.4)	13.4 (10.9)	4.1 (4.7)	19.8 (8.9)
Kerman (KER)	2.0 (2.9)	14.7 (11.1)	2.6 (2.6)	19.4 (9.1)
Khouzestan (KH)	3.1 (3.7)	8.0 (8.1)	0.5 (1.5)	11.8 (9.1)
Chaharmahal and Bakhtiari (CB)	1.3 (2.3)	12.4 (10.1)	4.5 (4.7)	18.2 (8.1)
Hormozgan (HO)	3.0 (3.4)	6.2 (8.9)	1.7 (2.5)	10.9 (9.3)
West Azerbaijan (WA)	4.2 (4.8)	14.1 (11.7)	0.9 (2.2)	19.2 (10.2)
Ardabil (AR)	2.4 (3.2)	14.9 (10.7)	2.4 (3.7)	19.7 (9.0)
Razavi Khorasan (RK)	3.5 (3.7)	5.1 (6.7)	5.1 (4.1)	13.6 (7.0)
Overall	**3.3 (4.6)**	**12.6 (10.4)**	**2.1 (3.4)**	**18.0 (9.5)**

### Socioeconomic inequality in DMF, D, M and F

Table 3 shows the estimated values of RC for DMF for the total sample and for each province separately. The findings suggested that DMFT was mainly concentrated among disadvantaged population in the 14 provinces included in the study (RC = − 0.064; 95% confidence interval (CI), − 0.066 to − 0.063). The estimated RC suggested statistically significant inequality in the DMF in favour of the rich in all 14 provinces. The extent of socioeconomic-related inequality in DMFT was found to be especially high and low in the provinces of Mazandaran (RC = − 0.1228), and Razavi Khorasan (RC = − 0.0327), respectively.

**Table 3:** The relative concentration index of DMFT score by total of sample and province

Cohort site	Relative concentration index	*p* value	95% confidence interval	
			Lower limit	Upper limit
Fars (FA)	−0.0590	< 0.001	− 0.0623	− 0.0556
Guilan (GI)	− 0.0599	< 0.001	− 0.0665	− 0.0534
Kermanshah (KSH)	− 0.0769	< 0.001	− 0.0831	− 0.0707
East Azerbaijan (EA)	− 0.0605	< 0.001	− 0.0642	− 0.0567
Mazandaran (MA)	− 0.1228	< 0.001	− 0.1282	− 0.1175
Sistan and Balouchestan (SB)	− 0.0402	< 0.001	− 0.0466	− 0.0338
Yazd (YA)	− 0.0536	< 0.001	− 0.0588	− 0.0484
Kerman (KER)	− 0.0748	< 0.001	− 0.0799	− 0.0696
Khouzestan (KH)	− 0.0851	< 0.001	− 0.0941	− 0.0760
Chaharmahal and Bakhtiari (CB)	−0.0696	< 0.001	− 0.0755	− 0.0636
Hormozgan (HO)	− 0.0787	< 0.001	− 0.0951	−0.0623
West Azerbaijan (WA)	−0.0749	< 0.001	− 0.0849	− 0.0648
Ardabil (AR)	− 0.0730	< 0.001	− 0.0785	−0.0675
Razavi Khorasan (RK)	−0.0327	< 0.001	− 0.0435	− 0.0219
Total	**− 0.0643**	**< 0.001**	**− 0.0660**	**− 0.0627**

**Fig. 1:**
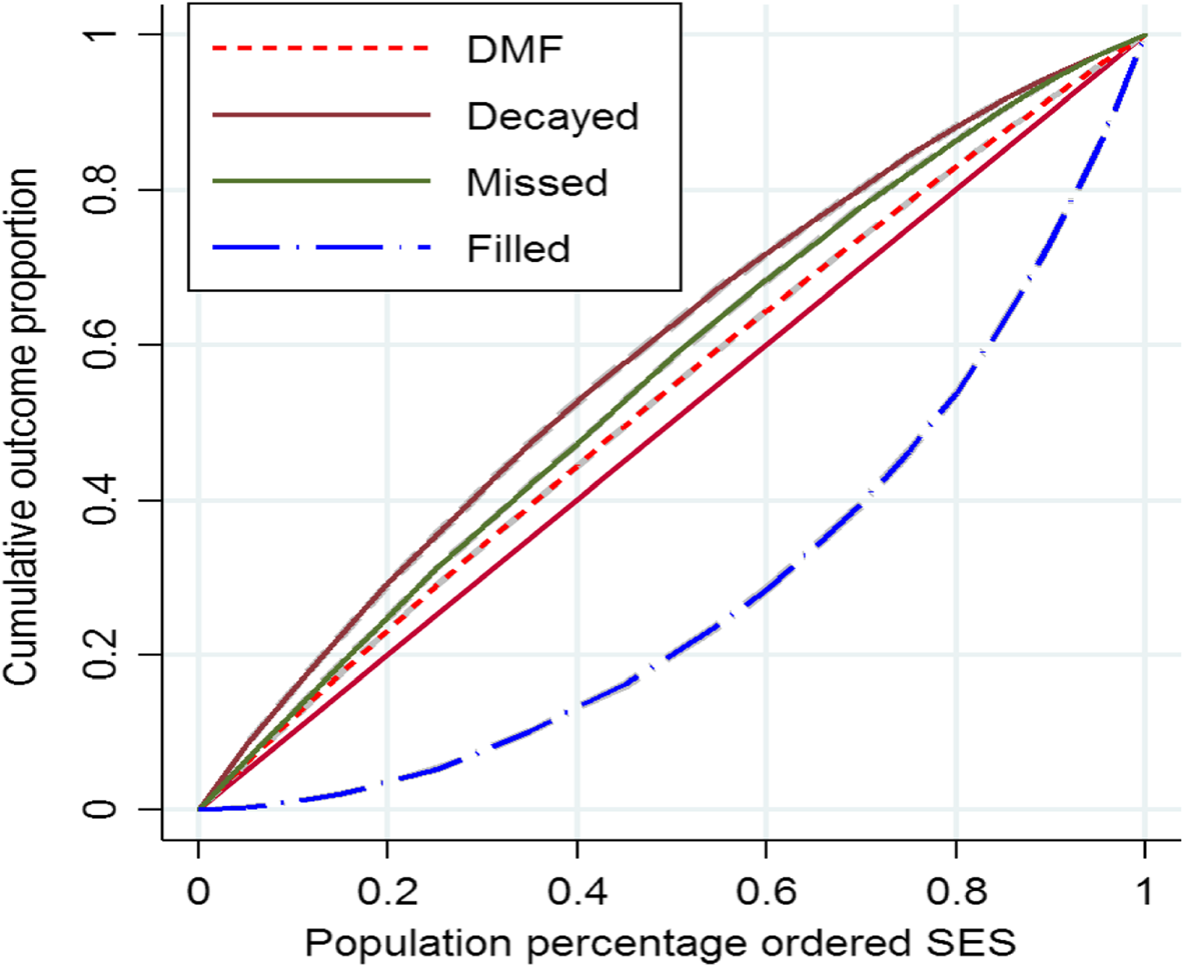
The concentration curve for D, M, F and DMFT for total samples

The RC of D, M and F for the total sample was estimated to be − 0.1684 [95% CI: − 0.1726 to − 0.1642], − 0.1086 [95% CI: − 0.1111 to − 0.1061] and 0.4028 [95% CI: 0.3982 to 0.4075], respectively. The concentration curve of D, M, F and DMFT for the total sample is showed in the Fig. 1. As illustrated in the Fig. 1, the concentration curve for D, M and DMFT is lies above the perfect line; suggesting a higher D, M and DMFT scores is more concentrated among the poor. Also, the concentration curve for F is lies below the perfect line and it is means that the higher F score is more prevalent among the socioeconomically advantaged population.

**Fig. 2:**
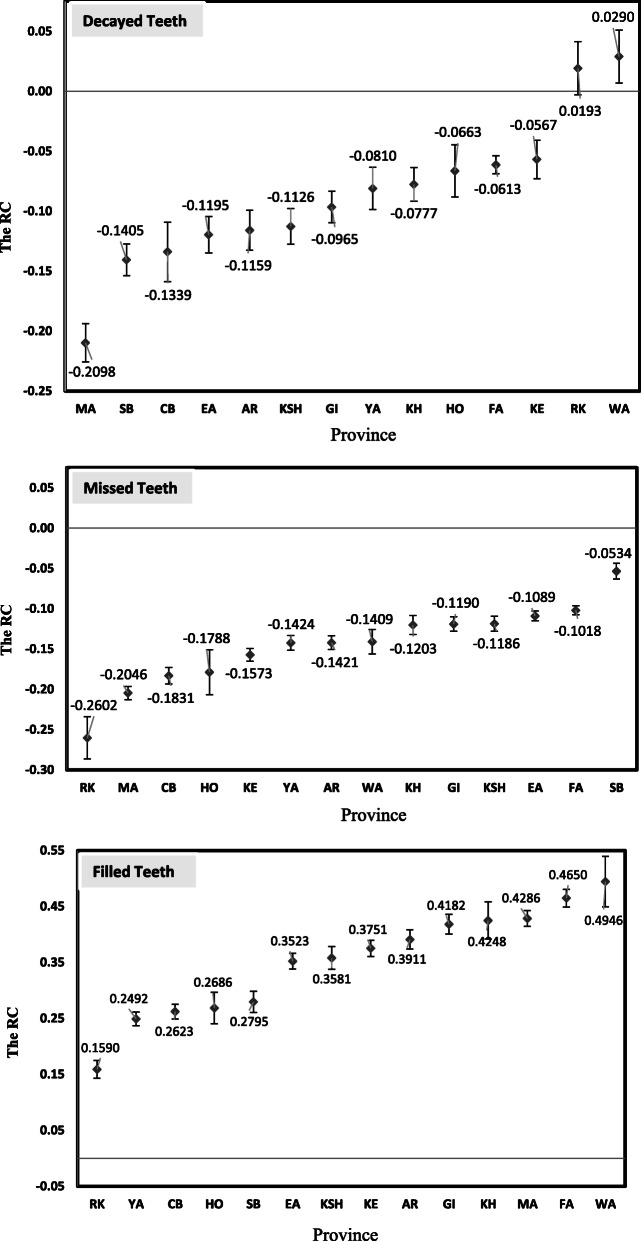
Socioeconomic-related inequalities in D, M and F teeth across 14 provinces in Iran. *Note:* with 95% confidence interval

Figure 2 shows the RC for D, M and F teeth for each province separately. As illustrated in the Fig. 2, the sign of RC for D and M teeth for all provinces, except for West Azerbaijan and Razavi Khorasan, is negative and statistically significant; suggesting a higher prevalent of D and M teeth among the poor. Also, the sign of RC for F teeth is positive and significant and it is indicates that the F teeth for all provinces is more concentrated among the rich.

### Determinants of socioeconomic inequalities in DMFT

Table 4 contains the results of the decomposition analysis of socioeconomic-related inequalities in DMFT measured for all included cohorts. The table reports 1) the coefficients estimating the effect of each explanatory factor on DMFT, 2) the elasticities of DMFT with respect to explanatory variables, 3) the RC for each explanatory variable, and 4) the contribution of each factor to the overall RC for DMFT.

**Table 4:** Decomposition of socioeconomic inequalities in DMTF in Iran

Variables	Coefficient	Elasticity	RCx	Absolute Contribution	% Contribution	Summed
Demographic variables						
35–44	ref					
45–54	4.071^*^	0.076	0.028	0.002	−3.3	
55–64	8.572^*^	0.115	−0.074	−0.008	13.2	
65 and older	11.311^*^	0.040	−0.216	−0.009	13.5	23.5
*Sex*						
Male	−1.137^*^	−0.028	0.107	−0.003	4.7	4.7
Females	ref					
*Marital status*						
Single	ref					
Married	1.644^*^	0.083	0.026	0.002	−3.3	
Divorced or widowed	1.770^*^	0.007	−0.285	−0.002	3.0	−0.3
Socioeconomic status variable						
1 (Poorest)	ref					
2	−1.014^*^	−0.011	−0.403	0.005	−7.1	
3	−1.835^*^	−0.020	−0.003	0.000	0.0	
4	−2.699^*^	−0.030	0.398	−0.012	18.7	
5 (Wealthiest)	−3.933^*^	−0.044	0.799	−0.035	54.7	66.3
Behavioral variables						
*Smoking status*						
Smoker	4.081^*^	0.049	0.047	0.002	−3.6	−3.6
Non-smoker	ref					
*Drinking alcohol*						
Yes	0.401^*^	0.002	0.203	0.000	−0.6	−0.6
No	ref					
Region (province)						
Fars (FA)	ref					
Guilan (GI)	−5.748^*^	−0.026	−0.209	0.005	−8.5	
Kermanshah (KSH)	−2.807^*^	−0.012	−0.100	0.001	−1.9	
East Azerbaijan (EA)	1.380^*^	0.009	0.019	0.000	0.0	
Mazandaran (MA)	−2.208^*^	−0.010	0.143	−0.001	2.2	
Sistan and Balouchestan (SB)	−2.596^*^	−0.009	0.023	0.000	0.0	
Yazd (YA)	0.994^*^	0.004	0.226	0.001	−1.4	
Kerman (KER)	−0.047	0.000	0.313	0.000	0.0	
Khouzestan (KH)	−8.165^*^	−0.032	−0.257	0.008	−12.7	
Chaharmahal and Bakhtiari (CB)	−0.636^*^	−0.002	0.472	−0.001	1.3	
Hormozgan (HO)	−8.372^*^	−0.012	−0.148	0.002	−2.8	
West Azerbaijan (WA)	−1.075^*^	−0.002	−0.140	0.000	0.0	
Ardabil (AR)	0.778^*^	0.003	0.256	0.001	−1.1	
Razavi Khorasan (RK)	−2.237^*^	−0.003	0.566	−0.002	2.5	−22.3
Sum				**−0.043**		**67.6**
Residual				**−0.021**		**32.4**
Total				**−0.064**		**100**

* *P*-value less than 0.05

Note: ref. = reference category in the analysis

The results of multivariable regression (the coefficients results) indicated that older age was associated with higher DMFT score. Compared to females, males had statistically significantly greater DMFT score. Also, the DMFT score among single was found to be lower than compared to other marital status groups. The mean of DMFT score was lower among people with better-off compared to socioeconomically disadvantaged individuals. Positive associations were found between unhealthy behaviors of smoking status and drinking alcohol and DMFT score. The results also suggested higher DMFT score among individuals residing in the provinces of GI, KSH, WA, RK, CB, KE, HO, KH, SB and MA than those living in FA province.

The RC for each of explanatory variables, *RC*_*k*_, were presented in the third column of Table 4. A positive value of this index suggested that the explanatory variable is more concentrated among the wealthier people and vice versa. The *RC*_*k*_ results indicated those who were male, married, smokers and drinker were relatively wealthier in the study population, whereas individuals who were divorced or widowed and older were relatively poor.

The term “contribution” shows how much the variation of each explanatory variable across SES groups can explain the observed association between SES and DMFT score. If the sign of contribution for a given explanatory factor is positive (negative), it suggests that the socioeconomic distribution of the factor and the association between this variable and DMFT score leads to a higher DMFT score among the worse-off (better-off). Based on the results reported in Table 2, it is evident that SES is the main factor that contributed to the concentration of DMFT score among the poor (66.3% calculated as its contribution divided by the total the contribution of SES/total RC). Besides socioeconomic status, demographic factors (age, gender and divorced or widowed) were the main factors contributed to the concentration of DMFT among lower SES groups in DMFT in the study population.

As reported in Table 4, 67.6% of socioeconomic-related inequality in DMFT was explained by the explanatory variables included in the study. The remaining 32.4% of the inequality in DMFT are associated with variables that are not included in the study.

## Discussion

Dental caries is a major oral health problem in developed and developing countries. The current studies [9–13] also highlighted socioeconomic inequalities in oral health problem (defined as differences in incidence or prevalence of oral disorders) across socioeconomic groups. Although inequality in dental caries continues to be a main oral and public health issue in Iran, there exist scant studies that aim to examine socioeconomic inequalities in oral health in Iran [4]. The aim of present cross-sectional study is to quantify the extent of socioeconomic-related inequality in DMFT among Iranian adults and to understand determinants of socioeconomic inequality in DMFT.

The average DMFT index was found to be 18.0 in 14 provinces in Iran with significant variation across provinces. We found statistically significant pro-rich inequality in DMFT score in all the provinces included in the study. Socioeconomic-related inequality in DMFT score was found to be large in provinces such as Ardabil, Yazd, Kerman, East Azarbaijan and Fars. A study by Moradi and collogues also indicated that the higher concentration of poor DMFT score among the poor in Kurdistan city, Iran [4]. A study conducted in Kosovo indicated that the mean of DMFT was 11.6 in the 35–44 year age group, 13.7 among the 45–64-year age group, 18 in the 65–74-year age group, and 23.19 in the age group of 75+ years [35]. The mean of DMFT among the 35–44 age groups was 16.1 in Germany [36], 15.4 in Hungary [37] and 14.7 in Austria [38]. However, the mean DMFT score in our study (18.0) was higher than as compared with the findings these studies that can be explained by this fact that the age of our samples (18–65) is greater than other studies.

Besides SES, our study also showed that being a female, older adults, married, smoking and drinking alcohol were associated with higher DMFT score among Iranian adults. Our study indicated that higher DMFT score among individuals residing in the cohorts of WA, AR, YA, KE, FA and EA compared to other provinces included in the study. A study by Piovesan et al. [39] also found higher DMFT scores among women compared to men. A study conducted by Ditmyer et al. [11] also indicated that higher DMFT scores among women and older individuals. Since the population of older adults in Iran is increasing, this finding calls for further attention to deliver oral health care in this population. Previous works also highlighted unhealthy behavior (e.g., drinking alcohol and smoking) as main determinants of oral health [39, 40]. One possible explanation of the effect of drinking on DMFT score is that alcohol users consume a high amount of refined carbohydrates and neglect both personal and professional health care, which, in turn, may lead to high DMFT score among these populations. In line with previous studies [41, 42], we found that higher DMFT score among smokers than non-smokers. Ueno et al. [43] have investigated that the association between active and passive smoking on oral health among adults in Japan. Their study demonstrated that active smoking as well as secondhand smoking may have negative effects on oral health. The decomposition results indicated that the SES itself is the main determinant of socioeconomic-related inequality in DMFT score in Iran. The negative effect of SES on DMFT score can be due to, for example, lower access of lower SES individuals to dental health care services compared to their higher SES counterparts. The inverse association between SES and oral health status is highly documented in previous studies. Moradi et al. found that individuals with lower SES had higher DMFT score [44]. Wang et al. investigated the association between SES and dental caries in older adults in China and concluded that household income and educational attainment were protective factors against dental caries [45]. A significant positive association between dental health status and a higher level of education was also observed in Mexico [46].

Beside SES, being male and older age and widow or divorced were the main factors contributing to the concentration of DMFT among the worse-off in Iran. The negative contribution of being male to socioeconomic inequality in DMFT is explained by the fact that men compared to women have lower DMFT score (see the negative elasticity reported for this variable in Table 2) and they are relatively better-off compared to women in Iran (see the positive *RC*_*k*_ for this variable Table 2). Older age and being window or divorced increase the concentration of DMFT score among the poor because older adults and those who are window or divorced in Iran have higher score of DMTF score (see the positive elasticity reported for these variables in Table 2) and they are relatively poor in Iran (see the negative *RC*_*k*_ for these two variables in Table 2).

The findings of the present study should be interpreted in light of some limitations. Firstly, since this study is a cross-sectional design, we were unable to establish causal relationships between explanatory variables and DMFT score in the decomposition analysis. Secondly, data for this study extracted from 14 provinces and just for adults (aged 35 years and above) in Iran; thus, the generalizability of our results to other provinces and other age groups is partially limited. Thirdly, the DMF score and its socioeconomic-related inequality can be influenced by other important factors such as ethnicity or nationality and living area (rural vs. urban area). These factors, however, were excluded from the study due to the lack of data in the dataset used in the present study.

## Conclusion

This study revealed that dental caries, as measured by DMTF score, was concentrated among socioeconomically disadvantaged adults in Iran. We also observed significant variations in socioeconomic inequality in DMTF score among different provinces in Iran. As our study demonstrated SES, being a male, older age and being a widow or divorced as the main factors contributing to the concentration of DMFT among the worse-off in Iran, it is recommended to focus in the oral health status of these groups in order to reduce socioeconomic inequality in oral health among adults in Iran. For example, as the existing studies (e.g., [47–50]) showed pro-rich inequalities in health care utilization in Iran, it is recommended to expand oral health care services for these groups through publicly funded primary health care in Iran. Moreover, it should be noted that reducing socioeconomic inequalities in dental caries should be accompanied by appropriate health promotion policies that focus actions on the fundamental SES causes of dental disease.

## Data Availability

All necessary data are presented within the manuscript. All other materials and data are available upon request.

## Appendix

The characteristics of cohort centers in Iran

**Table:**

Row	Province	Population*	Cohort site	Population*	Cohort population	Main Ethnicities
1	Ardabil	1,270,420	Ardabil	529,374	8192	Turk
2	Chaharmahal and Bakhtiari	947,763	Sharekord	93,104	6664	Lor
3	East Azerbaijan	3,909,652	Khameneh	3056	14,978	Turk, Azari
4	Fars	4,851,274	Kavar	31,711	2244	Fars, Turk
			Kharameh	18,477	10,662	Fars, Arab
			Fasa	110,825	10,113	Fars, Arab and Turk
5	Guilan	2,530,696	Some’e Sara	58,658	10,511	Gilaki
6	Hormozgan	1,776,415	Bandare Kong	19,213	3570	Arab
7	Kerman	3.164,718	Rafsanjan	161,909	9982	Fars
8	Kermanshah	1,952,434	Ravansar	47,657	10,077	Kurd
9	Khouzestan	4,710,506	Hoveizeh	19,481	9156	Arab
10	Mazandaran	3,283,582	Sari	309,820	10,253	Tabari
11	Razavi Khorasan	6,434,501	Mashhad	3,001,184	2189	Fars
			Sabzevar	243,700	784	Fars
12	Sistan and Balouchestan	2,775,014	Zahedan	587,730	8318	Balouch
13	West Azerbaijan	3,265,219	Ghoushchi	2787	3662	Turk, Azari
14	Yazd	1,138,533	Shahedieh, Yazd	18,309	9901	Fars

References: 1- Persian cohort sites, available from: http://persiancohort.com/cohortsites/, access: April 21, 2019. 2- Iran statistics center, available from: https://www.amar.org.ir, access: April 21, 2019

## Abbreviations

DMFT: Decayed, missing, and filled teeth
SES: Socioeconomic status
PERSIAN: Prospective Epidemiologic Research Study in IRaN
MoHME: Ministry of Health and Medical Education
PCA: Principal component analysis
RC: Relative concentration index
GC: Generalized (absolute) concentration index
