# Correction: Decomposing socioeconomic inequality in dental caries in Iran: cross-sectional results from the PERSIAN cohort study

**DOI:** 10.1186/s13690-022-00959-3

**Published:** 2022-09-26

**Authors:** Farid Najafi, Satar Rezaei, Mohammad Hajizadeh, Moslem Soofi, Yahya Salimi, Ali Kazemi Karyani, Shahin Soltani, Sina Ahmadi, Enayatollah Homaie Rad, Behzad Karami Matin, Yahya Pasdar, Behrooz Hamzeh, Mehdi Moradi Nazar, Ali Mohammadi, Hossein Poustchi, Nazgol Motamed-Gorji, Alireza Moslem, Ali Asghar Khaleghi, Mohammad Reza Fatthi, Javad Aghazadeh-Attari, Ali Ahmadi, Farhad Pourfarzi, Mohammad Hossein Somi, Mehrnoush Sohrab, Alireza Ansari-Moghadam, Farhad Edjtehadi, Ali Esmaeili, Farahnaz Joukar, Mohammad Hasan Lotfi, Teamur Aghamolaei, Saied Eslami, Seyed Hamid Reza Tabatabaee, Nader Saki, Ali Akbar Haghdost

**Affiliations:** 1 Research Center for Environmental Determinants of Health, Health Institute, Kermanshah University of Medical Sciences, Kermanshah, Iran; 2 School of Health Administration, Faculty of Health, Dalhousie University, Halifax, Canada; 3 Social Development and Health Promotion Research Center, Kermanshah University of Medical Sciences, Kermanshah, Iran; 4 Department of Social Welfare Management, University of Social Welfare and Rehabilitation Sciences, Tehran, Iran; 5 Social Determinants of Health Research Center, Guilan University of Medical Sciences, Rasht, Iran; 6 Department of Health Information Technology, Paramedical School, Kermanshah University of Medical Sciences, Kermanshah, Iran; 7 Liver and Pancreatobiliary Diseases Research Center, Digestive Diseases Research Institute, Tehran University of Medical Sciences, Tehran, Iran; 8 Department of Anesthesiology, Sabzevar University of Medical Sciences, Sabzevar, Iran; 9 Noncommunicable Diseases Research Center, Fasa University of Medical Sciences, Fasa, Iran; 10 Gastroenterohepatology Research Center, Shiraz University of Medical Sciences, Shiraz, Iran; 11 Social determinants of Health Research Center, Urmia Jundishapur University of Medical Sciences, Urmia, Iran; 12 Modeling in Health Research Center, Shahrekord University of Medical Sciences, Shahrekord, Iran; 13 Digestive Disease Research Center, Ardabil University of Medical Sciences, Ardabil, Iran; 14 Liver and Gastrointestinal Diseases Research Center, Tabriz University of Medical Sciences, Tabriz, Iran; 15 Diabetes Research cente, Mazandaran University of Medical Sciences, Sari, Iran; 16 Health Promotion Research Center, Zahedan Jundishapur University of Medical Sciences, Zahedan, Iran; 17 Department of Cardiology, Medical school, Rafsanjan University of Medical Sciences, Rafsanjan, Iran; 18 Gastrointestinal and Liver Diseases Research Center, Guilan University of Medical Sciences, Rasht, Iran; 19 Shahid Sadoghi University of Medical Sciences, Yazd, Iran; 20 Department of Public Health, School of Public Health, Hormozgan University of Medical Sciences, Bandar Abbas, Iran; 21 Pharmaceutical Research Center, Pharmaceutical Research Institute, Mashhad University of Medical Sciences, Mashhad, Iran; 22 Research Center for Health Sciences, Shiraz University of Medical Sciences, Shiraz, Iran; 23 Hearing Research Center, Ahvaz Jundishapur University of Medical Sciences, Ahvaz, Iran; 24 Modeling in Health Research Center, Institute for Futures Studies in Health, Kerman University of Medical Sciences, Kerman, Iran

**Correction to: Archives of Public Health 78, 75 (2020)**

10.1186/s13690-020-00457-4

Following publication of the original article [1], the authors reported an error of the ethical code in the Ethics approval and consent to participate section.

The original version was:

While each cohort center received the ethical approval from local universities, for the purpose of this study and pooling all PERSIAN data, the ethics committee of Kermanshah University of Medical Sciences approved the study (IR.KUMS.REC.1397.187).

The correct version is:

While each cohort center received the ethical approval from local universities, for the purpose of this study and pooling all PERSIAN data, the ethics committee of Kermanshah University of Medical Sciences approved the study (IR.KUMS.REC.1397.866).

The original article [1] has been corrected.
